# The virtual hospital as a means for undergraduate medical students to practice clinical reasoning: a qualitative interview study

**DOI:** 10.1186/s12909-026-09063-4

**Published:** 2026-03-30

**Authors:** Josefina Robertson, Frida Rydberg Antezana, Erika Tyrberg, Johan Westin, Matilda Liljedahl, Marie Studahl

**Affiliations:** 1 Department of Infectious Diseases, Institute of Biomedicine, Sahlgrenska Academy, University of Gothenburg, Gothenburg, Sweden; 2 Department of Infectious Diseases, Sahlgrenska University Hospital, Gothenburg, Region Västra Götaland, Sweden; 3 Department of Oncology, The Institute of Clinical Sciences, Sahlgrenska Academy, University of Gothenburg, Gothenburg, Sweden; 4 Department of Oncology, Sahlgrenska University Hospital, Gothenburg, Region Västra Götaland, Sweden

**Keywords:** Clinical education, Clinical reasoning, Interactive learning, Interview study, Qualitative methods, Undergraduate medical students, Virtual hospital, Virtual patients.

## Abstract

**Background:**

Entering the clinical environment presents medical students with several challenges. They need to understand the organization of a hospital ward and practice clinical decision making; that is, to learn clinical competencies. It may be beneficial for students to also practice clinical competencies in a simulated environment. The virtual hospital is a digital educational tool, in which 3-year undergraduate medical students meet, diagnose, and treat fictive patients suffering from infectious diseases. The students work with the virtual hospital in a seminar-like setting with other students under supervision of clinical teachers. Three group sessions are held during the course of one week where students can follow patient progress each day in real time and follow up on initiated treatments. The aim of this study was to explore experiences of the virtual hospital among students and clinical teachers.

**Methods:**

This qualitative interview study was set at an academic teaching hospital in Gothenburg, Sweden. Based on an interview guide, medical students (n=6) and clinical teachers (n=6) were interviewed. Interviews were transcribed verbatim and subject to qualitative content analysis.

**Results:**

Students experienced the virtual hospital to be highly educational, as well as realistic and engaging. They raised how open discussions were allowed and encouraged, and inherently student-centered. Teachers experienced the virtual hospital as having learning in focus and that the many disturbances of a regular clinical ward were absent. They found the virtual hospital to be user-friendly, instructive and enjoyable and that the teaching method in itself directed them into a student-centered manner.

**Conclusion:**

Both students and teachers were highly appreciative of using the virtual hospital as a learning activity and teaching method. The study suggests that the virtual hospital is suitable for students in the early clinical phase of medical education as it introduces clinical routines and practices in a controlled manner. The virtual hospital proved effective in introducing undergraduate medical students to clinical reasoning and may act as a complement to regular clinical rotations. There is great potential in the virtual hospital, as it is flexible and can be adapted to other medical areas and settings, other educational programs, and interprofessional education.

**Supplementary Information:**

The online version contains supplementary material available at 10.1186/s12909-026-09063-4.

## Introduction

Entering the clinical environment presents students with a number of challenges. Students are supposed to acquire clinical knowledge and skills within a, for them, previously unknown setting [1]. Also, the clinical phase of medical education requires students to learn other competencies than emphasized in the preclinical phase. Clinical reasoning, which is a fundamental and complex skill for health care professionals, involves critical thinking, interpreting information and problem-solving, as well as the application of medical knowledge to arrive at the best possible outcome for the patient [2]. Clinical reasoning has mainly been practiced during clinical rotations, but it may be beneficial for students to practice clinical reasoning also in simulated settings. It is also reported that utilizing various forms of educational strategies may promote medical students’ development of clinical reasoning [3].

For decades now, virtual patients (VPs) have been used to introduce clinical cases and support the development of clinical competencies among students, including clinical reasoning [4, 5]. The simulated setting holds opportunities for teachers to control the content of the medical knowledge provided in cases, and also the possibility to integrate theory with practice. Typically, VPs have been offered as complementary to traditional teaching, to assist students in their learning. However, it has also been shown that VPs are more successfully used as integrated into clinical courses. According to Edelbring et al., students who were asked to present and/or discuss the VPs with a teacher in a follow-up seminar perceived a higher benefit of VPs compared to students who were not offered any follow-up [6]. Lee et al. also suggested that VPs should include both pre- and post-activity with reflection and human feedback, in order to be effective [7]. It is therefore reasonable that virtual tools in education are implemented in an integrative way in courses instead of disconnected from regular teaching.

At the authors’ academic teaching hospital, a web-based virtual hospital teaching platform was developed in 2020, which simulates an in-patient ward with several admitted patients. The virtual hospital has been further developed since and is now an integrated part of the undergraduate medical training. Today, the fictive patient bank contains approximately 25 virtual cases with different infectious diseases. The virtual hospital platform is used for a combination of self-studies, and interactive sessions with a clinical teacher, extended over a three-day period. Patients can be followed from admission to the ward until discharge, which allows simulation of a complete disease course. The aim of this study was to explore experiences of the virtual hospital among students and clinical teachers.

## Methods

### Design and setting

**Fig. 1:**
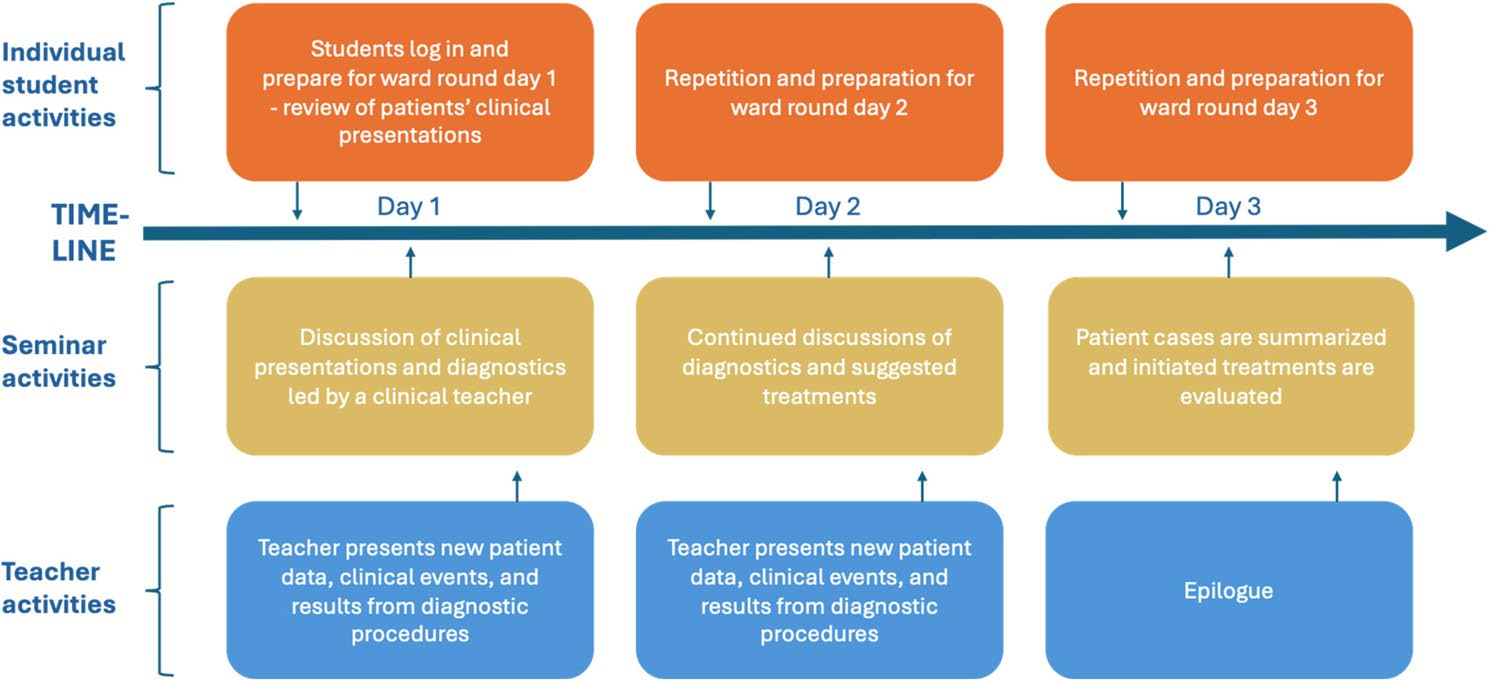
Timeline of student, seminar and teacher activities in the virtual hospital

Situated in a constructivist research tradition, this qualitative study was conducted with data collected through semi-structured individual interviews with students and teachers. The study was set at an academic teaching hospital in Gothenburg, Sweden. An integrated 20-week clinical course is given to 3rd Year undergraduate medical students which includes infectious diseases, venereology, immunology, microbiology, rheumatology, and allergology. The infectious diseases module comprises theoretical lectures and seminars, as well as clinical rotations at the in-patient infectious department. As part of the module, students can participate in seminars called “The virtual hospital” in groups of 10–15 students, led by one to two clinical teachers who are clinical specialists and lecturers in infectious diseases. The virtual hospital was initially developed during the Covid-19 pandemic as clinical rotations were restricted, but it is now part of the regular course. Seminars (2–3 h) are held three days in a row, comprising the same group of students and teacher(s) (Fig. 1).

**Fig. 2:**
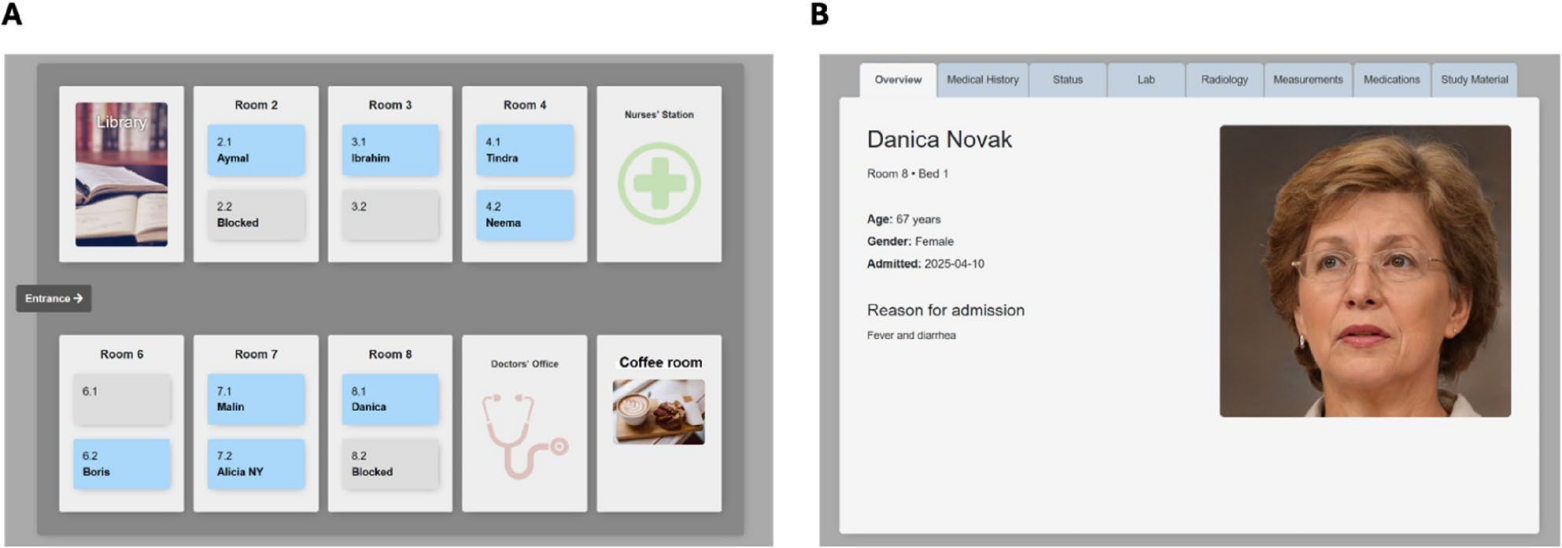
Overview of an infectious ward (**A**) and an individual patient record (**B**) in the virtual hospital (AI generated image by Generated Photos)

Before the first seminar, students can log into the virtual hospital platform and read about fictive patients admitted to an infectious ward. Available information about the patients include name, picture (AI generated by Generated Photos), age, sex, medical history and current drugs, results from physical examination, laboratory tests, and radiology (Fig. 2).

During the seminars, the clinical teacher(s) guides students in diagnosing and treating patients through asking questions, responding to students’ suggestions and relate the cases to theoretical and preclinical knowledge. At the first day’s seminar, the students discuss preliminary diagnosis and propose diagnostic procedures and clinical investigations, with support from teachers. At the next day’s seminar, new clinical information (e.g. lab results) about the patients is provided, and students may need to reconsider their initial diagnosis and/or treatment. At the third day’s seminar, the patient cases are summarized, and initial treatments are evaluated. In this way, students can follow patients’ clinical courses for a couple of days. The seminar’s interactive format allows for the teachers to adjust the content of the discussion based on identified knowledge gaps. As preparation for the next day’s seminar, the students may be given different tasks in order to gain in-depth knowledge on a specific topic. It is also possible for teachers to choose patients with uncommon, but important infectious diseases that are difficult to cover in a real clinical setting, to be included in the virtual hospital seminar. This can be arranged to make sure to meet the learning outcomes of the course. On request, the virtual hospital has been introduced also at other clinical courses and educational programs.

### Sampling and data collection

Students (*n* = 6) and clinical teachers (*n* = 6) with experience from the virtual hospital were invited face-to-face or by email to participate in an interview, representing a purposive sample. None declined participation. Clinical teachers (one male/five female) had extensive clinical experience ranging from four to 30 years. Interview guides were constructed, based on the aim of the study and the collective experience of the research group (Additional files 1 and 2). Face-to-face interviews were held by JR, FRA, ET and/or MS at a time and place of convenience for the participant (all participants chose to be interviewed in the clinical workplace) and performed by two researchers collaboratively, except for three interviews with students that were performed by one researcher due to logistical reasons. The interviewers were not known by the students before the interview, however, clinical teachers were familiar with the interviewer, as they are part of the same clinical context. For all participants, the explorative nature of the study was articulated and it was explicitly stated that no right or wrong answers existed. No one outside the research team was present during the interviews. Interviews lasted between 30 and 60 min. They were audio recorded and transcribed verbatim. Field notes during or after the interviews were not systematically taken, and therefore not considered as data in the study.

### Data analysis

Data were subject to inductive content analysis which is suitable to analyse human experiences [8]. Although content analysis can be performed with varying level of interpretation, analysis was here chosen to address the manifest content in data and the level of interpretation were regarded as relatively low, since the phenomenon under study has not been explored in any detail previously [9]. The data sets from students and clinical teachers were analyzed respectively as it was estimated that their experiences might differ from each other. Firstly, transcripts were read through. Secondly, text extracts representing meaning units were identified. Meaning units in each transcript were identified by at least two researchers independently to assure no possible meaning units were missing. Thirdly, meaning units were arranged into preliminary categories and themes through discussions in the research group. After having revisited the transcripts on several occasions, categories were finalized and related to each other and consensus on the final result was reached through discussion in the entire research group. For each data set (students and teachers), an overarching theme was identified, underpinned by two or three categories which in turn were built up by subcategories. The theme for each data set was considered as a central pattern in all categories and hence, communicate the results on a more abstract and interpretative level. No software tool was used in data analysis.

## Results

### Students’ perspectives

**Fig. 3:**
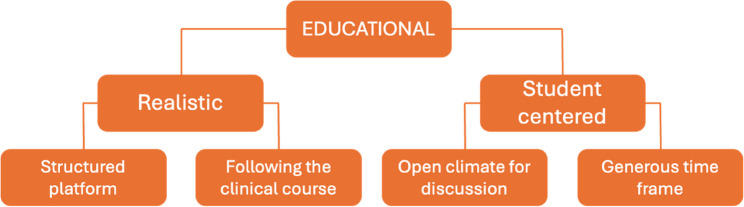
Overview of the students’ experiences of the virtual hospital

From the students’ perspective, one overarching theme with two categories divided into subcategories was identified (Fig. 3).

### Overarching theme: Educational

Collectively, the students experienced the virtual hospital and interactive learning as highly educational.

*"I would say that the virtual hospital is very educational. I have probably learned more in this part of the course than the previous ones."*

They appreciated the teaching format, as it invites discussion and allows students to receive direct feedback from the teachers.

*"Everything is case-based, we get concrete discussion questions and then we get continuous input from teachers. So I think it has been very educational."*

The virtual hospital assisted students in learning how to assess the severity of patients’ clinical presentations.

*“I got a better sense of what distinguishes a seriously ill patient from a moderately ill one.”*

Furthermore, students experienced that the session provided good preparation for interacting with real patients in the hospital.

*"Now I feel much more confident about how to approach patients with diseases that I saw in the virtual hospital."*

### Category: Realistic

The students expressed that the virtual hospital experience resembled the work of a real doctor, despite not being physically present in a hospital or interacting with real patients.

*"I haven’t been to a hospital yet, but it feels like this is how it works."*

They found the content clinically relevant, and the teaching format realistic, as it allowed for dynamic patient scenarios, with disease progression unfolding over several days.

*"This felt more alive in a way. You could expect that a patient might get worse overnight. They might need to be intubated."*

#### Subcategory: Structured Platform

The students felt that the structured layout of the platform provided them with a sense of how a clinical ward is organized, making it more realistic, which facilitated learning.

*"It’s easier to remember than just sitting and reading in a book."*

They also mentioned that navigating the platform was stimulating, as it allowed them to form a clear view of the patients’ current status.

*"It was fun to go into the different rooms and gather information."*

The virtual hospital was therefore perceived as both educational and realistic.

*"When I opened the virtual hospital, and saw that it was divided into rooms. There were isolation rooms… it felt quite real in a way."*

#### Subcategory: Following clinical course

The students expressed that it was valuable to follow a patient’s disease progression, with new information provided daily, just as it would happen in reality.

*"This is more realistic… you see a patient and follow the entire course of their illness."*

*"You got the feeling that it was like a morning round. Things could happen during the night, and we were told that something acute had occurred."*

The students also appreciated the opportunity to return to the same patient over several days, maintaining a continuous dialogue with the teachers. This was seen as facilitating learning compared to more traditional case-based learning, which does not follow a patient’s condition over time.

*"I think the virtual [hospital] was better because of the continuity; you repeat the same case over several days."*

### Category: Student centered

The students appreciated that the teachers in the virtual hospital actively invited discussion and ensured that all students had the opportunity to speak. They felt that the teachers were focused on their learning and genuinely cared about helping them learn as much as possible.

*"They were very committed. It felt like they wanted us to learn."*

#### Subcategory: Open climate for discussion

Students felt that the teaching environment fostered an open climate, encouraging active participation from all students in the assessment of patients.

*"There are many who don’t usually talk who have spoken in these exercises."*

Several students expressed that they dared to say things that they were not entirely sure about and that in this way they could learn from each other.

*"I like thinking out loud. I thought that was a good aspect—that it was okay to think out loud and to make mistakes."*

*"It felt like we had a very flowing discussion and a permissive atmosphere."*

#### Subcategory: Generous time frame

The students appreciated that the teaching environment was relaxed, and that the ample time dedicated to each patient contributed to a stimulating learning atmosphere.

*"I also learn better when I feel good. I think it’s fun."*

They also expressed that the virtual hospital provided the opportunity to delve deeper into challenging topics.

*"I learned a lot. It’s completely stress-free and without pressure, which makes learning easier."*

### Clinical teachers’ perspectives

**Fig. 4:**
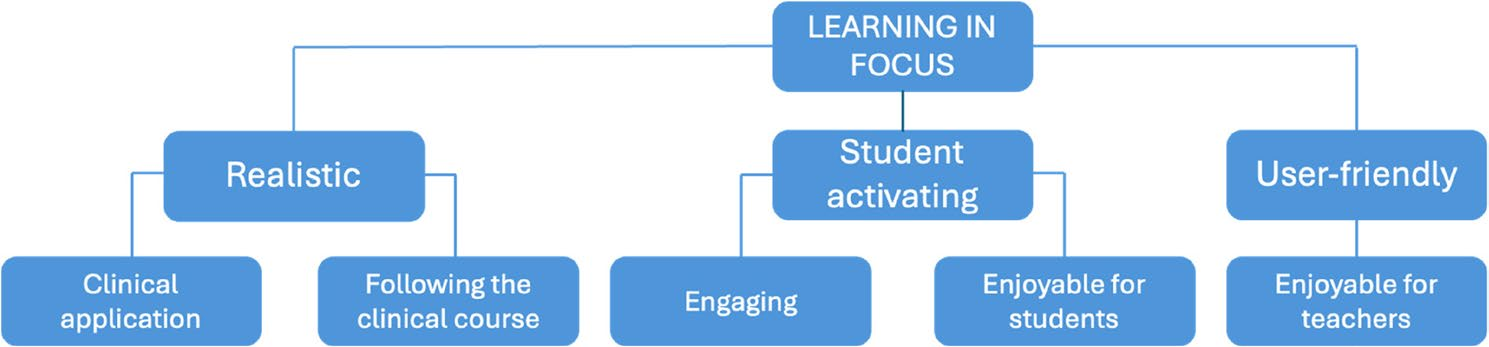
Overview of the clinical teachers’ experiences of the virtual hospital

From the clinical teachers’ perspective, one overarching theme (learning in focus) with three categories and thereto related subcategories was identified (Fig. 4).

### Overarching theme: Learning in focus

Teachers felt that the virtual hospital provided an opportunity for the students’ learning experience to be in focus, giving them space and attention, in contrast to the typical ward rounds, where both the needs of patients and clinical staff must be considered.

*“There’s a lot of distractions in the ward. There are nurses*,* and… where are the rooms*,* and here’s a sluice room. It’s much more streamlined here; you can focus on our key teaching points and some internal medicine. But the interruptions are gone—the phone ringing or any of the other distractions typical of a ward.”*

The teachers thus considered the virtual hospital to be a very good complement to regular clinical rotations, as it allows students to take center stage.

*“The students get more out of the round than a regular round. Now there was an opportunity where they were really involved from start to finish*,* and everything is about them understanding what is happening with the patients. How to think about treatment*,* differential diagnoses*,* cultures*,* and things like that.”*

However, the teachers agreed that the virtual hospital could not fully replace clinical rotations but should be seen as a complement.

*“This emotional thing*,* it is not present. That part is somewhat missing*,* and they don’t engage in ethical thinking in the same way. In reality*,* you always have that with you when interacting with patients.”*

### Category: Realistic

The teachers perceived the virtual hospital as resembling real-life practice in ways that traditional clinical case seminars could not replicate. Since the platform is structured like a medical record, they felt it mimics how one works in reality, where information is gathered from various sources such as medical records, lab results, and imaging.

*"This way of thinking, how we actually work, how we do things in everyday practice, this is what we do in the wards."*

Additionally, the teachers believed that the ability to follow multiple patients simultaneously also made the virtual hospital more realistic than traditional clinical case seminars.

*"And the fact that there are many patients at the same time, each with different conditions, is also very good."*

#### Subcategory: Clinical application

The teachers described that students get the opportunity to apply their theoretical knowledge in a clinical setting.

*“They gain a more clinical and reality-based insight into their knowledge.” *

Teachers also felt that students were compelled to think ahead regarding the next steps in diagnosis and treatment.

*“Here, they are forced to think ahead. And I think that is highly developmental.”*

The teachers also challenged students to make decisions about how to proceed with patient care.

"I think there’s a point in having a certain time pressure. They have to make up their mind, they are forced to work in a focused way."

The teachers believed this approach better prepares students for the future when they will independently manage patients, as they learn to extract relevant information from the medical histories and train their memory skills.

"This mindset, how we work… […] all these pieces of the puzzle must be gathered by themselves to form a picture of the patient."

#### Subcategory: Following the clinical course

The teachers felt that the virtual hospital very well illustrates the clinical course, as students can follow patients over several days. The teachers described how this setup allows information to be repeated, that they can refer back to previous discussions, which was perceived as beneficial from a learning perspective. The ability to follow the clinical course also allowed for unexpected developments that could alter the patient’s case and management.

*"Oh, now he had a fever of 40 degrees, but that’s unfortunate; I didn’t expect that. He was supposed to go home, and then you change your mind. And that’s a good medical strategy—being able to absorb new information and change course. And I think that’s well illustrated in the virtual hospital."*

The teachers described the virtual hospital as an introduction for students to clinical thinking, where they continuously receive more information and may need to revise their previous assessments.

"It’s not just exactly what the textbook says—sometimes there’s a twist in the cases, which is a lot of fun."

### Category: Student activating

The teachers described how the platform makes it easy to engage students in active learning.

"It’s impossible to use it without activating the students."

The teachers felt that students were forced to think more independently by being tasked with summarizing, reasoning, and presenting patient cases, and by being challenged with questions.

"They act like junior doctors who are responsible in some way— they are forced to find the answers themselves and think independently."

"They are given the opportunity to explain why they want to do certain things."

The teachers also believed the teaching format encouraged students to discuss cases among themselves, creating an environment where more students dare to speak up.

#### Subcategory: Engaging

The teachers perceived the virtual hospital as highly engaging for the students, who immerse themselves in the role of the doctor and in the patients’ situations. The realistic design, featuring patient photos, medical records, lab results, and imaging, contributed to students entering more into the role of a physician. They also experienced that the interaction between students and teachers further promoted engagement and involvement.

*"The students felt that we—teachers and students—were truly part of the round. We were involved from start to finish. Sometimes, when students are on a clinical rotation, they may only join at the end of a patient’s care, which doesn’t provide a clear continuity of care."*

#### Subcategory: Enjoyable for students

The teachers felt that students found the virtual hospital enjoyable, which facilitated learning.

*"One of the students said, I can’t believe we don’t have the virtual hospital every semester. And it was very positive that they thought it should be included in all their other courses as well. So, I think it added more than we expected."*

### Category: User-friendly

The teachers found the virtual hospital platform to be user-friendly. They did not need any formal introduction and could use the platform immediately.

*"The platform was very intuitive."*

They also expressed that the platform provided clear guidance on how each patient case should proceed, which allowed for flexibility in teaching.

"[The seminar] gets a great structure, that makes it very…you talk about what you should talk about."

The teachers appreciated that the platform included prepared questions with suggested answers.

"From a teacher’s perspective, it creates a safe environment because we know what questions are coming, so to speak. Someone has already thought through the answers."

"You feel secure as a teacher."

#### Subcategory: Enjoyable for teachers

The teachers found the virtual hospital experience enjoyable.

*"I really liked the teaching method. It felt exciting."*

This was partly because it was a new way of teaching, distinct from their previous teaching methods.

*"I thought it was really fun; it’s different from anything we’ve done in previous sessions."*

They also expressed that their positive experience was enhanced by seeing the students’ engagement and positive reactions.

*"...then you could see that they were tired because they had learned something new. […] I interpreted that as it had not been very boring, but it had really been a mental effort."*

## Discussion

This study explored students’ and teachers’ experiences of using the virtual hospital in the setting of a clinical course in infectious diseases. Both students and teachers were highly appreciative of using the virtual hospital as a learning activity and teaching method. Students found the virtual hospital to be highly educational, providing new insights into the clinical environment as it was experienced as realistic and engaging. Also, students appreciated the interactive and student-centered features of the virtual hospital where open discussions were allowed and encouraged. Teachers emphasised how the virtual hospital allowed learning to be the focus of students’ attention, as many of the regular disturbances of a clinical ward were absent. They found the virtual hospital to be user-friendly, instructive and enjoyable and that the teaching method in itself directed them into a student-centered manner. It was obvious that the interactions between teacher and students were significant in shaping student´s experiences supporting the notion that VP´s are most successfully integrated into regular teaching [7]. No major differences between students’ and teachers’ experiences were found, but as expected they held slightly different perspectives.

A major advantage of the virtual hospital seemed to be that it was experienced as a realistic setting, especially as the virtual hospital was organized as a clinical round with several patients to care for simultaneously. Following the patient’s clinical course added to the realistic experience with new clinical data being presented and patients’ conditions improving or deteriorating just like in reality. Compared to traditional case seminars, it can be argued that the realistic experience here enabled students to try out the role of a clinician rounding a ward. Both students and teachers emphasized how the virtual hospital became emotionally engaging as they felt they were truly a part of the ward round. It is possible that this assisted in students’ learning as the emotional engagement made students care about patients and made the experience stimulating and motivating. The authentic experience can also assist students in the transfer of knowledge and skills to upcoming clinical rotations [10]. Despite efforts to design the virtual hospital as realistic as possible, it is important to note that a fictive patient on a screen can never truly replace a real patient with a physical body and emotions and the possibility to interact with the patient as human being.

The clinical environment is known to be unpredictable and full of disturbances. Patient care commonly needs to be the primary priority and learning therefore assumes second place [11]. Moreover, clinical teachers often report limited time for teaching [12]. In essence, the clinical environment is designed first and foremost for patients. For students, in their early clinical learning process, the clinical environment can be overwhelming and elusive, making learning difficult [1]. By contrast, the virtual hospital enabled a realistic clinical environment without disturbances and distractions. It is reasonably that this enabled learning to be in focus and the students’ needs to be in the center of attention without competition from patients, as illustrated in the theme *Learning in focus*.

The virtual hospital seemed to provide space, time, and an allowing environment for students to try out ideas and discuss clinical cases. The interactive design of the virtual hospital where the VP´s were discussed in a physical seminar allowed for both feedback from teachers and peers, something that is supported in previous research [7, 13]. Students were in the virtual hospital demanded to integrate clinical data and arrive at a decision, even if the entire picture of the patient’s presentation was not yet clear. In that sense, the virtual hospital offered possibilities for students to be introduced to the practice of clinical reasoning in a safe and supporting environment and within the presence of an engaged teacher, something that is encouraged in the literature [3]. An advantage of the virtual hospital is that clinical information is presented gradually during the course of the week, known as a serial-que approach [14]. This approach simulates a real clinical setting where a patient presents with symptoms and treatments need to be initiated e.g. before all lab results have arrived. Compared to traditional case seminars, which often are of whole-case format, it can be argued that students here were given the possibility to practice clinical reasoning through the serial-que approach [14]. Students needed to make treatment decisions for patients, although they are only in their third year of training. In that sense, they were acting as junior doctors, however, without the risk of patient harm. For students to be able to start ‘acting’ as junior doctors can be a way to stimulate the development of their professional identity as future physicians [15]. However, compared with traditional case seminars, the virtual hospital demanded a higher level of engagement from both students and teachers, which can be a challenge. Additionally, the discussions could in the virtual hospital take unexpected directions based on students’ knowledge gaps which complicated time management.

From the teachers’ perspective, the virtual hospital was found to be easily managed and intuitive to follow. They hence emphasized how it made teaching enjoyable and effortless. The possibility to control and adjust the content depending on students’ needs and previous experience may be important for teachers to be able to address all learning outcomes in the course and focus on relevant issues. There may also be a potential for less experienced clinical teachers to develop as teachers through the use of the virtual hospital, as teachers felt secure in this teaching method.

In the virtual hospital, the teachers were very much involved in supporting and guiding students in their work with the fictive patients, although students also worked independently from teachers at times. This is important, as it has been shown previously that guidance and support from teachers is vital when students use virtual patients as a learning activity [7].

To develop an educational tool such as the virtual hospital demands time from both clinical teachers as well as individuals with programming competence. The virtual hospital also requires scheduled time for clinical teachers and students three days in a row, which may be challenging. However, once in place, the virtual hospital is easily managed and requires only minor adjustments when e.g. a new diagnostic tool is available or when teachers identify a need for a new patient case. Ultimately, the effort made in developing the virtual hospital is rewarded through the access to a valuable and maintenance free educational tool.

### Methodological considerations and reflexivity

This study holds both strengths and weaknesses. In line with the constructivist orientation of the study, data was considered to be generated in collaboration with the participants and analysis was performed from a subjectivist point-of-view [16]. The members of the research all have both academic and clinical training, and all were female, except for JW who is male. Several of the researchers were part of the initiative of the virtual hospital and developed both the virtual platform and the educational content. The involvement in development and implementation of the virtual hospital as an educational tool may have influenced the interpretation of data. The inclusion of a health professions education researcher (ML) who joined the team solitary for the research process was important here, to assist the team in challenging interpretations and bringing an outsider perspective. However, it may that the research process has been influenced by potential biases due to the research groups’ involvement in the virtual hospital. It was a strength that the analysis process was conducted collaboratively with high involvement from the entire research group as this allowed us to hold open and intense conversations about the data and the emerging results. Six students and six clinical teachers in the study were included. At the time for data collection, there were no more clinical teachers involved in the virtual hospital. It was choosen not to interview any more students, to leave time for thorough data analysis, however, the inclusion of more students may have brought perspectives on the virtual hospital not captured in the current study. Data saturation and member checking was not employed in the study. The study was set at a single institution, meaning that it may represent experiences specific for this institution. It may be beneficial to implement and evaluate the virtual hospital also in other settings. The COREQ checklist was used in reporting the study (Additional file 3).

## Conclusion

The virtual hospital proved to be a realistic and engaging way for students to learn clinical reasoning without the disturbances the clinical environment often presents. Students therefore highlighted how it was highly educational. For teachers, the virtual hospital was found to be user-friendly, instructive, and enjoyable, and the teaching method allowed for students’ learning to be in focus.

In conclusion, the virtual hospital seems suitable for students in the early clinical phase of medical education as it introduces clinical routines and practices in a controlled manner. The virtual hospital can therefore act as an important complement to regular clinical rotations to assist students in the transition into their new role as clinicians.

Moreover, the virtual hospital holds substantial potential for further development. It may be used in later phases of clinical training, where students can take a more independent role. It can also be introduced in other medical areas or in an out-patient setting. It can focus not only on strictly medical knowledge but also on other important areas of professional development, such as ethical considerations. Additionally, the virtual hospital may be used in other educational programs and in interprofessional learning.

## Supplementary Information

Supplementary Material 1.

Supplementary Material 2.

Supplementary Material 3.

## Data Availability

Data and materials are available from the first authors on reasonable request.
