# Supplementary material for: The virtual hospital as a means for undergraduate medical students to practice clinical reasoning: a qualitative interview study
Source: BMC Med Educ. 2026 Mar 30;26:616. doi: 10.1186/s12909-026-09063-4 (PMC13081561; doi:10.1186/s12909-026-09063-4)
Supplement: Supplementary file 1 — Supplementary Material 1. [file 12909_2026_9063_MOESM1_ESM.docx]

**INTERVIEW GUIDE: STUDENTS**

**IMPLEMENTATION**

- How many students were in your group?
- How was the teaching conducted using the virtual hospital? Were breakout groups used, and if so, how?
- Did you have one or two teachers? If you experienced both, which did you prefer? Please explain.
- What was it like to participate in the virtual hospital?

**LEARNING**

- What did you feel you learned?
- Was there anything that surprised you during the virtual hospital teaching?
- Did you notice any specific knowledge or insight you gained during the three days of the virtual hospital?
- Was there anything particular about the structure of the virtual hospital that facilitated your learning?
- Were you assigned to read preparatory material or complete preparatory tasks?
- If so, was this followed up the next day?
- Were you given responsibility for your own patient? Did you conduct rounds or summarize a patient case at any point?
- How was it for you to take responsibility for a patient?
- How did the group collaboration work out?
- What do you think is the optimal group size?
- Were there any disadvantages to the virtual hospital?
- How did you experience the support from the teacher(s) during the session?
- Can you give an example of something particularly good that the teacher(s) did or said?
- If you participated in traditional case-based teaching – in what way does the virtual hospital differ from that?
- Was there any difference in your and your classmates’ engagement and participation in the virtual hospital compared to case-based teaching?

**LOOKING AHEAD**

- Is there any feature you feel is missing in the virtual hospital?
- Do you see any specific technical development opportunities for the virtual hospital?
- Do you see any development opportunities in how the cases are structured?
- Do you see any specific content-related development opportunities for the virtual hospital?
- Do you see any development opportunities in the pedagogy, i.e., how the teachers guide the sessions?
- Is there anything you’d like to add? Would you like to participate in the virtual hospital again as a student?
