# Supplementary material for: The virtual hospital as a means for undergraduate medical students to practice clinical reasoning: a qualitative interview study
Source: BMC Med Educ. 2026 Mar 30;26:616. doi: 10.1186/s12909-026-09063-4 (PMC13081561; doi:10.1186/s12909-026-09063-4)
Supplement: Supplementary file 2 — Supplementary Material 2. [file 12909_2026_9063_MOESM2_ESM.docx]

**INTERVIEW GUIDE: CLINICAL TEACHERS**

**IMPLEMENTATION**

- Did you conduct the teaching session alone or together with another instructor?
- How did you practically carry out the teaching using the virtual hospital?
- Did you use breakout groups (small group discussions), and if so, how?
- Did you have the students read preparatory material or assign any “homework”?
- How was this followed up the next day?
- Did the students take responsibility for their own patient?
- Did the students conduct rounds with their own patient?
- Did the students present a summary of a patient?
- What was the size of your group, and what advantages and disadvantages do you see with that group size? What is the optimal group size? Is there a maximum number of students?
- Do you have any comments on the use of tabs, e.g., lab, radiology, medication?
- In what way did you use the tabs?

**PEDAGOGY**

- What are your thoughts on the concept – what can the virtual hospital contribute compared to traditional case-based teaching?
- What can the virtual hospital contribute compared to on-site clinical placement?
- What is the main advantage of structuring the teaching in this way?
- Do you see any disadvantages or weaknesses with this approach?
- What do you think is important for students to take away from the experience?
- What do you think the virtual hospital contributes to student learning?
- Did you notice any specific knowledge or insight that students gained during the virtual hospital session?

**THE TEACHING ROLE**

- How has it been for you personally to supervise in the virtual hospital?
- What preparations did you make?
- How did the collaboration with the other teacher work?
- Have you taught alone at any point? If so, how did it go?
- Did the division of labor between the teachers change, and if so, in what way?
- Had you agreed in advance on exactly how to teach?
- Did you do anything differently this term compared to previous terms?
- Do you perceive any difference in student engagement and participation in the virtual hospital compared to traditional case-based teaching?
- Comparing traditional case-based teaching with the virtual hospital – what do you think are the pedagogical differences?

**LOOKING AHEAD**

- Do you see any opportunities for improvement in the software’s workflow?
- Is there any feature you feel is missing in the virtual hospital?
- Do you see any specific technical development opportunities for the virtual hospital?
- Do you see any specific opportunities for content development in the virtual hospital?
- Do you see any potential for development in the pedagogy (interaction between teacher and student), in how we supervise students and deliver the educational component?
- Is there anything you would like to add?
